# Effects of different land use types on soil microorganisms and physicochemical properties in the Huaihe River source region

**DOI:** 10.1186/s12866-026-05035-2

**Published:** 2026-04-11

**Authors:** Xiaolong Hao, Hongen Liu, Tong Niu, Zhen Liu, Yanmei Wang, Xiaodong Geng, Qifei Cai, Juan Wang, Yongyu Ren, Fangming Liu, Zhi Li, Li Dai

**Affiliations:** 1 College of Forestry, Henan Agricultural University, Zhengzhou, 450046 China; 2 National Forestry and Grassland Administration Key Laboratory for Central Plains Forest Resources Cultivation, Henan Agricultural University, Zhengzhou, 450046 China; 3 Henan Province Engineering Technology Research Center for Idesia, Zhengzhou, 450046 China; 4 Resources and Environment College of Henan Agricultural University, Zhengzhou, 450046 China; 5 Key Laboratory of Farmland Quality Conservation in Huang-Huai-Hai Plain, Ministry of Agriculture and Rural Affairs, Zhengzhou, 450046 People’s Republic of China; 6 Key Laboratory of Soil Pollution Prevention, Control and Remediation in Henan Province, Zhengzhou, 450046 China

**Keywords:** Soil microorganisms, Land use types, Soil physicochemical properties, Soil microbial diversity, Huaihe River source region

## Abstract

To investigate the effects of different land‑use practices on the soil ecosystem in the Huaihe River source region, this study selected forestland, farmland, and nursery land in Tongbai County, Henan Province, as research objects. A combination of high‑throughput sequencing and soil physicochemical analysis was employed to systematically compare the soil microbial community structure, diversity, and their relationships with key environmental factors among the three land‑use types. The results showed significant differences in soil physicochemical properties: farmland had the highest contents of available phosphorus (AP) and available potassium (AK), forestland had significantly higher AP content than nursery land (*P <* 0.05), while nursery land exhibited significantly higher soil water content (SWC) and bulk density (SBD) than the other two land‑use types (*P <* 0.05). Microbial diversity analysis revealed that the bacterial Simpson index and the fungal Shannon and Simpson indices were relatively higher in farmland soil (*P <* 0.05). Redundancy analysis indicated that soil organic matter (SOM) was the key environmental factor driving changes in both bacterial and fungal community structures. In terms of community composition, Acidobacteriota and Ascomycota were the dominant phyla for bacteria and fungi, respectively. Functional prediction analysis further revealed that the biosynthesis pathway was the most prominent metabolic function in both microbial communities. In summary, land‑use practices significantly altered the soil physicochemical properties and microbial communities in the Huaihe River source region. Moderate agricultural use maintained relatively high microbial diversity, and its community structure was mainly regulated by soil organic matter. This study provides a scientific basis for understanding soil ecological processes and the sustainable management of land resources in this region.

## Highlights of the thesis

1. Quantitatively reveal the differential impacts of three typical land uses on soil nutrients and physical properties in the source region of the Huaihe River.
2. Studies have found that moderate agricultural use can maintain high underground microbial diversity and promote the differentiation of community structures among different land use types.
3. The study clearly indicates that soil organic matter (SOM) is a key environmental factor driving the reconstruction of bacterial and fungal communities, and provides statistical evidence.
4. The study reveals the characteristics of the core metabolic functions of soil microorganisms in the source region of the Huaihe River: functional prediction indicates that biosynthetic pathways are dominant in both bacteria and fungi, providing a new perspective for understanding the mechanisms of microorganism-mediated material cycling.

## Background

Land use refers to the development and utilization of land resources by humans based on the natural attributes of soil, under specific socio-economic and technological conditions, to meet developmental needs [1]. Different land-use types correspond to distinct vegetation types, management practices, and soil ecosystems, thereby exerting markedly different impacts on the soil environment [2–4]. Such influences profoundly alter the supply and cycling processes of soil nutrients and further drive the evolution of soil texture and below-ground microbial community structure [5, 6]. Long-term agricultural activities (e.g., fertilization, tillage) have been shown to significantly alter soil physicochemical properties [7, 8], whereas high-intensity disturbances (e.g., over-cultivation) may lead to soil structure degradation, nutrient loss, and microbial community decline [9]. Conversely, appropriate soil management practices (e.g., deep loosening, organic amendments) can improve soil structure, enhance nutrient availability, and promote the recovery of microbial diversity [10]. Soil microorganisms are an essential component of soil ecosystems [11], driving key ecological processes such as organic matter decomposition, nutrient cycling, and energy flow [12–14]. Their community composition and diversity are not only important indicators for assessing soil fertility and ecosystem health [15, 16], but also serve as a critical foundation for the sustainable development of soil ecosystems [17, 18]. Among them, bacteria, as the most abundant and functionally diverse group of soil microorganisms, play irreplaceable roles in carbon and nitrogen transformation, disease suppression, and plant growth regulation [19–23]. The composition and distribution of soil bacterial communities are influenced by soil physicochemical properties and environmental conditions, exhibiting distinct regional specificity [24]. Fungi, on the other hand, form symbiotic or parasitic relationships with plant roots and efficiently degrade complex organic matter [25], exerting a decisive influence on soil structure formation, nutrient mobilization, and ecosystem stability [26–29]. Notably, the structure and function of soil microbial communities are not static; they show high sensitivity and adaptability to environmental factors, especially soil physicochemical properties shaped by land-use practices, such as pH, organic matter, and nutrient availability. Both soil texture improvement and plant growth and development are influenced by soil fungal communities [30, 31]. Therefore, elucidating the response mechanisms of soil microbial communities under different land-use types is of key importance for understanding ecosystem functions and guiding sustainable land management [32].

The Huaihe River is one of China's important water systems, and the environmental quality of its source region (located in Tongbai County, Nanyang City, Henan Province) is directly linked to the water resource security, economic development, and cultural heritage of the entire basin. As a key interface for material exchange between terrestrial ecosystems and river systems, soil health directly influences the nutrient load and water quality entering the river. However, existing research on this ecologically sensitive zone of the Huaihe River source has primarily focused on hydrology or vegetation. Systematic studies on the structure and function of soil microbial communities under different land-use types, as well as their interactions with soil physicochemical properties, remain scarce.This study selected forested areas, farmland, and nurseries within the Huaihe River headwaters region. Employing high-throughput sequencing and multivariate statistical methods, it aims to elucidate the effects of different land-use practices on soil physicochemical properties and microbial community structure and function. Furthermore, it seeks to decipher the relationships between key environmental factors and microbial communities, thereby providing scientific evidence for soil health assessment and sustainable land resource utilisation in this area.

## Methods

### Overview of the research area

**Fig. 1:**
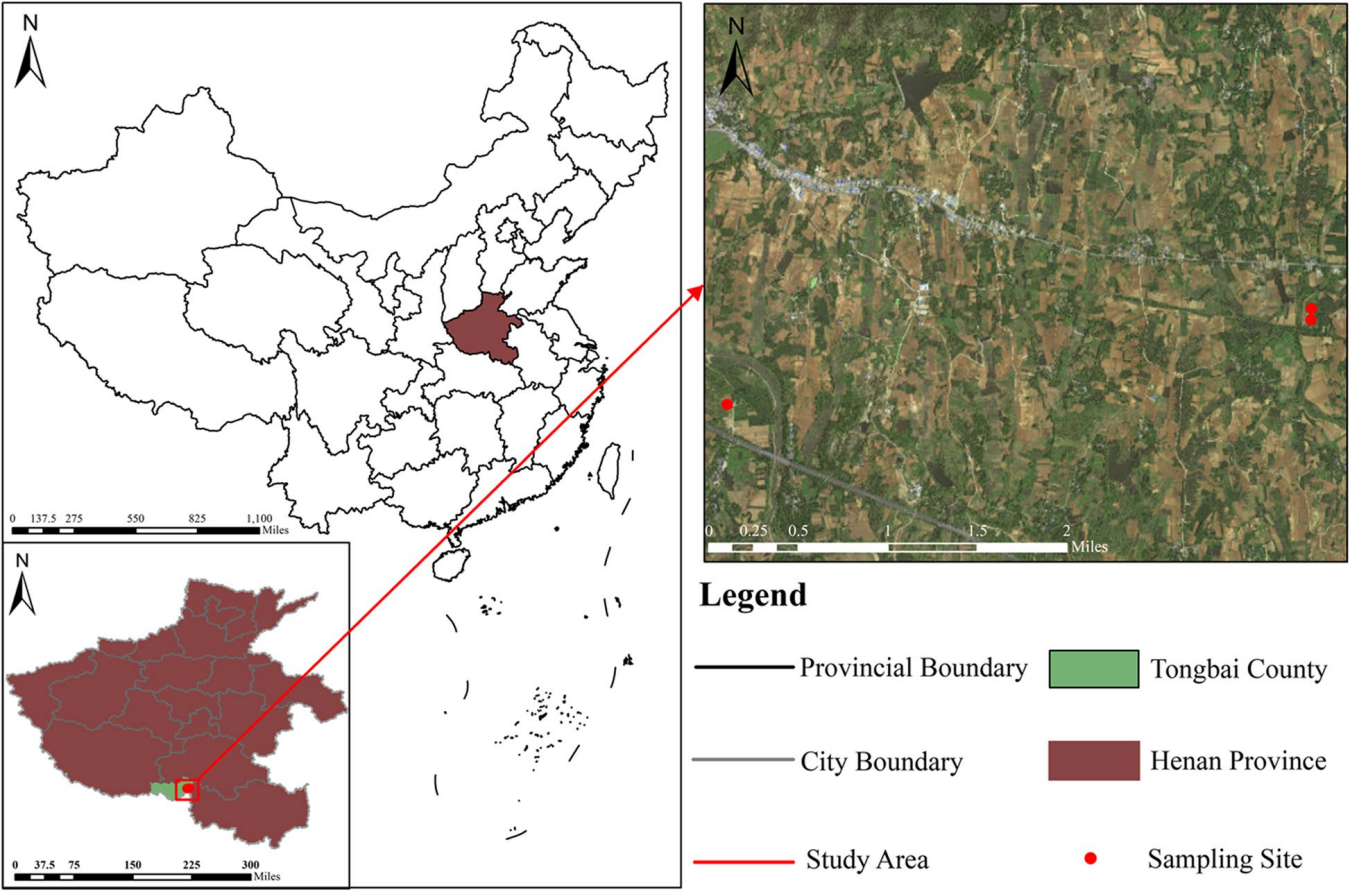
Schematic diagram of the location of the sampling point area

The study area is situated in Tongbai County, Nanyang city, Henan Province, which encompasses the source region of the Huaihe River Basin (Fig. 1). The Huaihe River originates from the valley northwest of Mount Taibai in the Tongbai Mountains, Tongbai County, Henan Province. It traverses multiple provinces, including Henan, Anhui, and Jiangsu, with a drainage basin spanning approximately 270,000 km^2^. Tongbai County features terrain dominated by low hills and gentle mountains, with elevations decreasing from northwest to southeast. The fertile soils developed in this transitional climatic zone between northern and southern China, characterized by a monsoon-influenced continental semihumid climate that blends subtropical and warm-temperate attributes. The average elevation ranges from 200–300 m. Climatic data indicate an annual mean temperature of 15℃ and relatively high precipitation levels, with annual rainfall between 933 and 1,181 mm. Tongbai County features rich vegetation resources, with a relatively high forest coverage rate. The region hosts multiple forest nature reserves, reflecting its significant ecological conservation value. Three representative different land use types were selected in the area, namely, forestland (LD), nursery land (MPD), and farm land (NT), of which the LD is a pure forest of perennial Chongyang wood with an average diameter at breast height of 35.67 cm and an average tree height of 16.5 m; the MPD is a plot that has been plowed over after seedlings have emerged from the ground; and the NT land is planted with wheat, peanuts, and maize in recent years.(Meteorological data sources: http://www.luoning.gov.cn/zjln/).

### Soil sample collection and preservation

Soil samples were collected in March 2024, with no precipitation recorded in the week prior to sampling. After removing surface litter, five sampling points were arranged in an ‘S’‑shaped pattern within each of the three study plots, resulting in a total of 15 soil samples. A stainless‑steel soil auger with a volume of 100 cm^3^ was used to collect soil from the 0–30 cm depth. After collection, the soil from each plot was homogenized and subdivided into three subsamples using the quartering method, yielding a total of nine subsamples. The subsamples were promptly placed in dry ice for low‑temperature preservation, transported to the laboratory, and stored at –80 ℃ for subsequent analysis. One portion was used for soil high‑throughput sequencing, and the other portion was used for determination of soil physicochemical properties.

### Determination of soil physical and chemical properties

Soil pH was measured potentiometrically. Exactly 10.00 g of air-dried soil passed through a 2-mm sieve was mixed with 25 mL of CO₂-free deionized water at a soil-to-water ratio of 1:2.5. The suspension was shaken for 30 min and then allowed to stand. The supernatant was measured with a pH meter (LC-PH-3S, Shanghai Lichen Bangxi Instrument Equipment Co., Ltd., Shanghai, China), and each sample was measured in triplicate. Soil water content (SWC) was determined by the oven-drying method. Approximately 100 g of fresh soil was placed in a pre-weighed aluminum box and dried to constant weight in an oven at 105 °C for 8–12 h. The moisture content was calculated from the mass difference before and after drying. Soil bulk density (SBD) was measured using the ring-knife method. Undisturbed soil cores were collected with a 100-cm^3^ ring knife. The soil together with the ring knife was oven-dried at 105 °C to constant weight, and the bulk density was calculated from the dry soil mass and the ring-knife volume. Soil organic matter (SOM) was determined by the potassium dichromate oxidation–external heating method. Exactly 0.1000 g of air-dried soil passed through a 0.15-mm sieve was mixed with 5 mL of 0.8 mol·L⁻^1^ potassium dichromate solution and 5 mL of concentrated sulfuric acid. The mixture was heated in an oil bath at 170–180 °C for 5 min. After cooling, the sample was titrated with a ferrous sulfate standard solution using phenanthroline as an indicator. Alkali-hydrolyzable nitrogen (AN) was measured by the alkali-diffusion method. A 2.00 g aliquot of air-dried soil was placed in the outer chamber of a diffusion dish, treated with 1.8 mol·L⁻^1^ sodium hydroxide solution, and incubated at 40 °C for 24 h in a constant-temperature incubator. The ammonia released was absorbed by boric acid in the inner chamber and then titrated with a 0.01 mol·L⁻^1^ hydrochloric acid standard solution. Available phosphorus (AP) was analyzed by the sodium bicarbonate extraction–molybdenum-antimony anti-colorimetric method. A 2.50 g portion of air-dried soil was extracted with 50 mL of 0.5 mol·L⁻^1^ sodium bicarbonate extractant (pH 8.5) by shaking for 30 min and then filtered. The filtrate was mixed with molybdenum-antimony anti-colorimetric reagent, and the absorbance was measured at 880 nm. Available potassium (AK) was determined by the ammonium acetate extraction–flame photometry method. A 5.00 g sample of air-dried soil was extracted with 50 mL of 1 mol·L⁻^1^ ammonium acetate extractant (pH 7.0) by shaking for 30 min and then filtered. The filtrate was analyzed for potassium ion concentration using a flame photometer (FP640, Shanghai Yidian Analytical Instrument Co., Ltd., Shanghai, China) [33].

### DNA extraction and high-throughput sequencing

After removal from the freezer (three replicates per treatment, nine samples in total), 0.1–0.5 g of each sample was accurately weighed, placed in a centrifuge tube containing extraction lysis buffer, and ground. Nucleic acids were extracted from the pretreated samples using the MagBeads Fast DNA Kit for Soil (MP Biomedicals, Irvine, CA, USA). The extracted DNA was checked for fragment size by 0.8% agarose gel electrophoresis and quantified with a Nanodrop spectrophotometer. The V3–V4 region of the bacterial 16S rRNA gene was amplified by PCR for sequencing using the primer pair F: ACTCCTACGGGAGGCAGCA and R: GGACTACHVGGGTWTCTAAT. For fungal analysis, the ITS1 region (~ 280 bp) was amplified with the primers ITS5 (GGAAGTAAAAGTCGTAACAAGG) and ITS2 (GCTGCGTTCTTCATCGATGC). The PCR program consisted of initial denaturation at 98 ℃ for 5 min; followed by 28 cycles of 98 ℃ for 30 s, 55 ℃ for 45 s, and 72 ℃ for 45 s; and a final extension at 72 ℃ for 5 min, after which the products were held at 12 ℃. Amplification products were verified by 2% agarose gel electrophoresis, and target bands were excised and purified using the Axygen Gel Recovery Kit (New York, NY, USA). Libraries were constructed with the Illumina TruSeq Nano DNA LT Library Prep Kit (San Diego, CA, USA) and sequenced on an Illumina NovaSeq 6000 platform using the NovaSeq 6000 SP Reagent Kit (500 cycles) in 2 × 250 bp paired‑end mode. Sequencing was performed by Personal Biotechnology Co., Ltd. (Shanghai, China).

### Data processing and analysis

Data analysis was performed using a combination of bioinformatic and statistical software. Raw sequencing data were processed using QIIME 2 (version 2022.11). Briefly, demultiplexed sequences were quality-filtered, primers were trimmed using cutadapt, and amplicon sequence variants (ASVs) were generated with the DADA2 plugin (error rate: 2 for both forward and reverse reads; truncation length: 223 and 230). Taxonomy was assigned against the SILVA 138 1 99% database. Microbial community composition at phylum and genus levels was visualized within QIIME 2 based on relative abundance. Alpha diversity indices (Shannon, Simpson, Chao1) were calculated in QIIME 2. Beta diversity was assessed using Bray–Curtis distances, and principal coordinate analysis (PCoA) was performed using the ape package in R (version 3.6.1). Venn diagrams were generated using the VennDiagram R package. Differential abundance analysis was conducted with LEfSe via the Python LEfSe package, and results were visualized using ggtree in R. Functional prediction of microbial communities was performed using PICRUSt2, and metabolic pathways were annotated against the MetaCyc database. Redundancy analysis (RDA) and correlation heatmaps were generated using the ropls and ggplot2 packages in R to explore relationships between microbial communities and soil properties. Basic data organization was performed in Microsoft Excel 2019. One-way ANOVA of soil physicochemical properties, alpha diversity indices, and metabolic pathway abundances was conducted using SPSS 27.0. Figures were prepared using Origin 2024 and R/ggplot2.

## Results

### Analysis of differences in soil physical and chemical properties among different land use types

A comparison of the soil physicochemical properties among the three different land-use types (Table 1) revealed no significant differences in soil pH, organic matter (SOM) content, or alkaline-hydrolyzable nitrogen (AN) content (*P* > 0.05). The pH and AN content in forestland (LD) were slightly higher than those in the other two types, while the SOM content was highest under farmland (NT) use. The available phosphorus (AP) in both farmland (NT) and forestland (LD) was significantly higher than that in nursery land (MPD) (*P <* 0.05). Available potassium (AK) showed a significant advantage in nursery land (MPD) and farmland (NT) (*P <* 0.05) and was significantly higher than in forestland (LD). Soil water content (SWC) and soil bulk density (SBD) were significantly greater in nursery land (MPD) than in the other two vegetation-use types (*P <* 0.05).

**Table 1:** Soil physicochemical properties of different land use types

Indices	MPD	NT	LD
pH	7.17 ± 0.09a	7.17 ± 0.17a	7.19 ± 0.10a
SOM g/kg^−1^	12.24 ± 4.54a	18.71 ± 7.38a	17.74 ± 3.62a
AN mg/kg^−1^	63.75 ± 16.49a	68.7 ± 19.95a	86.02 ± 33.92a
AP mg/kg^−1^	16.88 ± 7.49b	64.92 ± 8.56a	40.65 ± 5.25a
AK mg/kg^−1^	76.03 ± 5.23a	72.79 ± 5.56a	52.83 ± 11.18b
SWC/%	22.05 ± 0.32a	14.09 ± 2.04b	11.42 ± 1.47b
SBD g/cm^3^	1.25 ± 0.12a	1.13 ± 0.10ab	0.95 ± 0.10b

Exegesis: The data are the means ± standard deviations (*n* = 3), and different lowercase letters indicate significant differences in indicator values between different land use types (*P <* 0.05). The maximum mean is marked as a. MPD stands for nursery land, NT stands for farmland, and LD stands for forestland

### Analysis of the ASVs distributions of the soil microbial communities under the different land use types

**Fig. 2:**
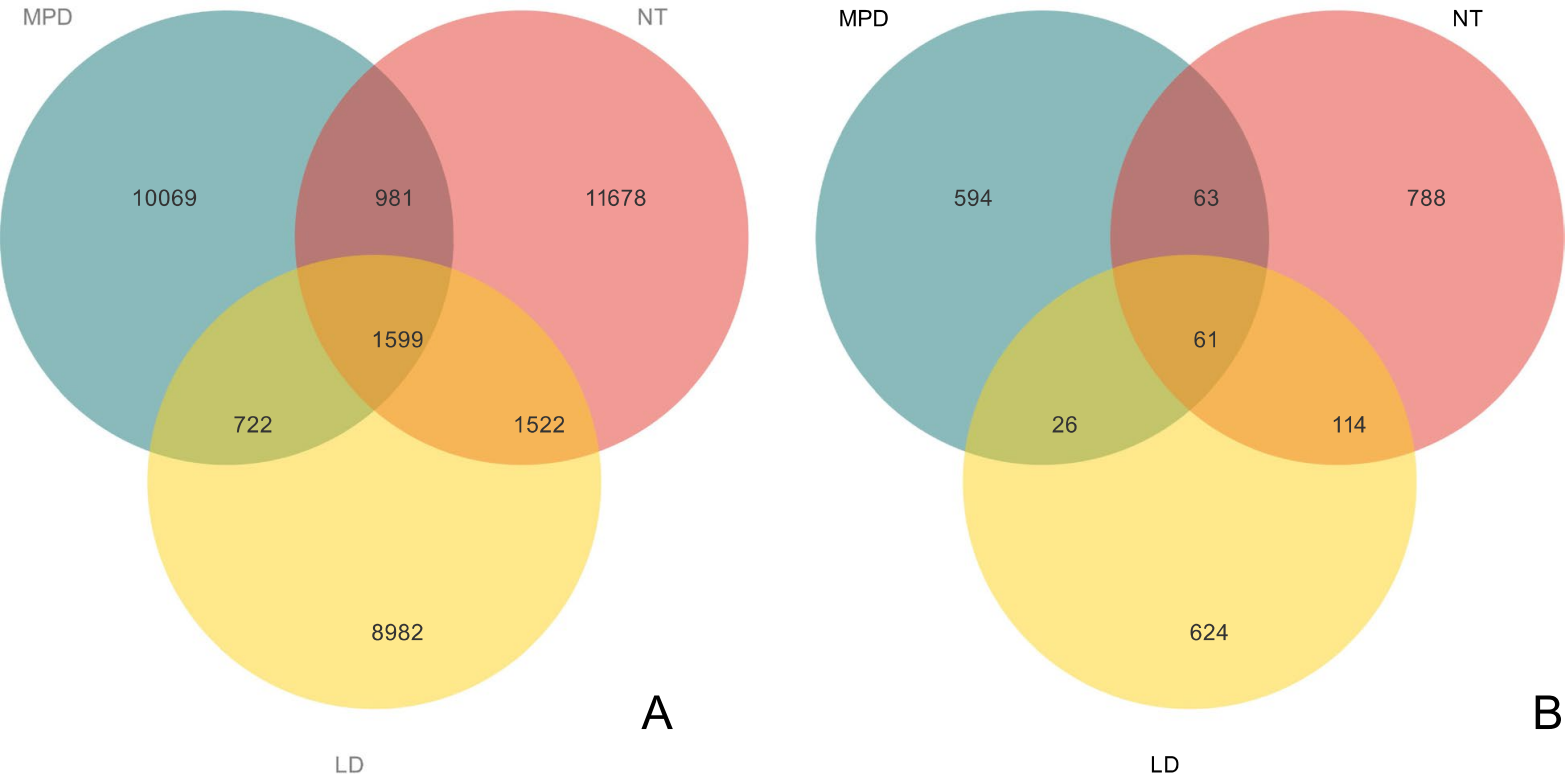
Distribution of the number of soil bacterial (**A**) and soil fungal (**B**) ASVs in different land use types. MPD stands for nursery land, NT stands for farm land, and LD stands for forestland

Following sequencing and ASV clustering analysis of soil microorganisms from the three different land-use types (Fig. 1), high-throughput sequencing of the soil samples yielded a total of 1,115,523 valid bacterial sequences and 752,649 high-quality sequences, with sequence lengths primarily ranging from 239 to 444 bp. For soil fungi, 1,260,648 raw sequences were obtained. After removing low-quality sequences and denoising, 1,091,981 valid sequences were retained, and 932,949 high-quality sequences were obtained following chimera removal, with sequence lengths distributed between 228 and 397 bp. As shown in Fig. 2A, a total of 35,553 bacterial ASVs (Amplicon Sequence Variants) were detected across the three land-use types, while 2,270 fungal ASVs were identified (Fig. 2B). The number of bacterial ASVs under different land-use types followed the pattern: farmland (NT) > nursery land (MPD) > forestland (LD). The number of ASVs shared by all three land-use types was 1,599. Between the different land-use types, farmland (NT) and forestland (LD) shared the highest number of ASVs, reaching 3,121. The numbers of ASVs unique to farmland (NT), nursery land (MPD), and forestland (LD) were 11,678, 10,069, and 8,982, respectively, accounting for approximately 32.85%, 28.32%, and 25.26% of the total bacterial ASVs.

The distribution of fungal ASVs counts across land-use types was: farmland (NT) > forestland (LD) > nursery land (MPD). Additionally, the number of ASVs shared by all three land-use types was 61. The numbers of ASVs unique to farmland (NT), forestland (LD), and nursery land (MPD) were 788, 624, and 594, respectively, constituting approximately 34.71%, 27.49%, and 26.17% of the total fungal ASVs.

### The impact of different land use types on the alpha diversity of the soil microbial community

In the analysis of alpha diversity of the soil bacterial community, the Shannon and Simpson indices were used to reflect diversity levels, and the Chao1 index was used to reflect species richness levels. As shown in Table 2, there were no significant differences in the Shannon or Chao1 indices among the different land‑use types (*P* > 0.05), although both indices showed the highest values under farmland use. In contrast, a significant difference was observed in the Simpson index between farmland and nursery land (*P <* 0.05).

**Table 2:** Analysis of the α diversity of soil bacteria in different land use types

Vegetation type	Chao1 index	Shannon index	Simpson index
MPD	5269.24 ± 540.50a	11.14 ± 0.19a	0.9990 ± 0.0002b
NT	6347.31 ± 571.16a	11.64 ± 0.19a	0.9994 ± 0.0001a
LD	5394.99 ± 953.37a	11.23 ± 0.34a	0.9991 ± 0.0002ab

Exegesis: The data are the means ± standard deviations (*n* = 3), and different lowercase letters indicate significant differences in indicator values between different land use types (*P <* 0.05). The maximum mean is marked as a. MPD stands for nursery land, NT stands for farmland, and LD stands for forestland

A comparative analysis of soil fungal community alpha diversity across different land-use types (Table 3) revealed significant differences among them (*P <* 0.05). The Shannon and Simpson indices were significantly higher in farmland (NT) and forestland (LD) than in nursery land (MPD) (*P <* 0.05). In contrast, there were no significant differences in the Chao1 index among the three land-use types (*P* > 0.05), although the Chao1 index in farmland (NT) was the highest among the three vegetation utilization types.

**Table 3:** Analysis of the α diversity of soil fungi in different land use types

Vegetation type	Chao1 index	Shannon index	Simpson index
MPD	290.39 ± 54.73a	4.71 ± 0.17b	0.8909 ± 0.0229b
NT	416.52 ± 103.51a	5.82 ± 0.64a	0.9495 ± 0.0310a
LD	325.83 ± 94.20a	5.70 ± 0.49a	0.9567 ± 0.0177a

Exegesis: The data are the means ± standard deviations (*n* = 3), and different lowercase letters indicate significant differences in indicator values between different land use types (*P <* 0.05). The maximum mean is marked as a. MPD stands for nursery land, NT stands for farmland, and LD stands for forestland

### Comparison of the soil microbial community structures of different land use types

#### Comparison of the soil bacterial community structure across different land use types

**Fig. 3:**
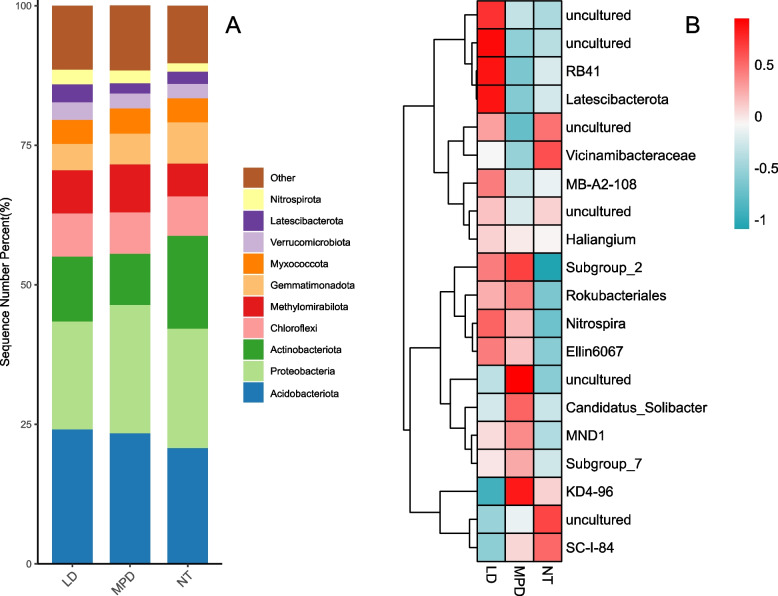
Soil bacteria at the (**A**) phylum level and (**B**) genus level in different land use types

The bacterial ASVs sequences obtained from the three sets of soil samples were delineated and assigned to 1088 genera in 43 phyla. When the sequences were analyzed and compared, the phylum whose relative abundance was ranked in the top 10 was selected for analysis (Fig. 3A). At the bacterial phylum level, the dominant phylum was Acidobacteria, with the highest percentage of 20.74% ~ 24.08%, Proteobacteria, with 19.30% ~ 23.01% of the total relative abundance value, and Actinobacteria, with 9.16% ~ 16.65% of the total relative abundance value. The distribution proportions of these dominant phyla vary among different land use types. Specifically, Acidobacteriota and Proteobacteria presented relatively high distributions in nursery land (MPD), accounting for 23.35 ~ 23.01% of the total relative abundance, which was the highest among the three land use types. In contrast, Actinobacteriota had the lowest proportion in nursery land (MPD), at only 9.16% among all land use types, while it reached the highest proportion of 16.65% in the NT land use type.

Cluster analysis of the top 20 most abundant bacterial genera across the three land-use types (Fig. 3B) revealed that while the bacterial community structures presented certain similarities, the dominance patterns of specific genera varied significantly among the different land-use regimes. Among the top 20 genera in terms of bacterial relative abundance, the vast majority had relatively high relative abundances under forestland (LD) conditions. Specifically, the following genera exhibited particularly prominent relative abundances: the uncultured genera in Desulfobacterota, the uncultured genera in Chloroflexi, the *RB41* genus in Acidobacteriota, and the *Latescibacterota* genus in Latescibacterota. In nursery land (MPD), the genera *Subgroup_2* and uncultured within Acidobacteriota, as well as the genus *KD4-96* within Chloroflexi, presented relatively high abundances. In the farmland (NT) land-use type, the relative abundances of the uncultured genus and *Vicinamibacteraceae* genus in Acidobacteriota, the uncultured genus in Gemmatimonadota, and the *SC-I-84* genus in Proteobacteria were greater than those in the other two land-use types.

MPD stands for nursery land, NT stands for farm land, and LD stands for forestland. In Fig. 3B, the uncultured genera are listed from top to bottom as follows (at the lowest clearly distinguishable taxonomic level): 1. Uncultured genus within the phylum Desulfobacterota; 2. Uncultured genus within the family Roseiflexaceae; 3. Uncultured genus within the order Vicinamibacterales; 4. Uncultured genus within the order Gaiellales; 5. Uncultured genus within the order Acidobacteriales; uncultured genus within the order Acidobacteriales; 6. uncultured genus within the family Gemmatimonadaceae.

#### Comparison of soil fungal community structure under different land use types

**Fig. 4:**
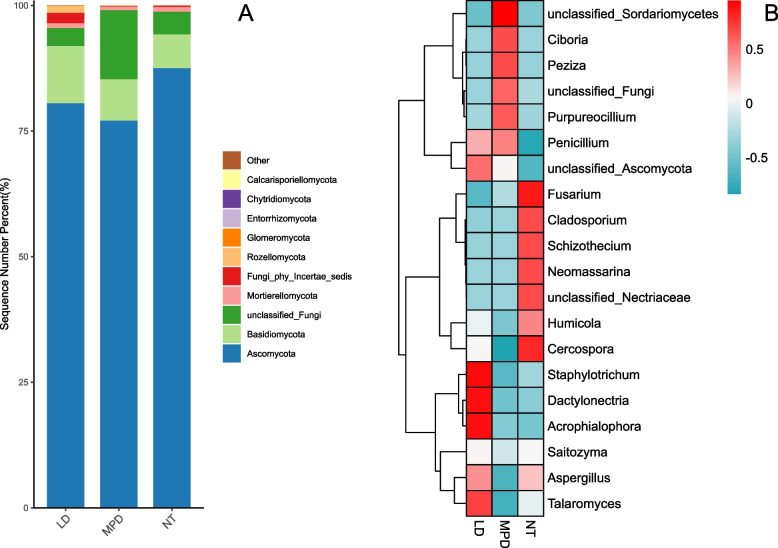
Soil fungi at the (**A**) phylum level and (**B**) genus level in different land use types. MPD stands for nursery land, NT stands for farm land, and LD stands for forestland

The fungal ASVs sequences obtained from the three soil samples were clustered into 14 phyla and 533 genera. For comparative analysis, the top 10 phyla by relative abundance were selected for analysis (Fig. 4 A). At the phylum level, the dominant phyla were Ascomycota (comprising 77.04% ~ 87.45% of the total relative abundance), Basidiomycota (6.77% ~ 11.37%), and unclassified fungi (3.61% ~ 13.73%). The relative abundances of the dominant phyla varied across different land use types. In the farmland (NT) land-use type, Ascomycota presented the highest relative abundance, accounting for 87.45% of the total. For Basidiomycota, the highest relative abundance (11.37%) was observed in the forestland (LD) land use type, which was nearly twice that of farmland (NT). The unclassified fungi phylum in nursery land (MPD) accounted for approximately 13.73%, which was approximately fourfold greater than those in forestland (LD) and farmland (NT).

Clustering analysis of the top 20 fungal genera by relative abundance across the three land use types (Fig. 4B) revealed that fungal communities in different land use types presented both structural similarities and distinct differences in dominance. In nursery land (MPD), higher relative abundances were observed for the genera unclassified_Sordariomycetes, *Ciboria*, *Peziza*, unclassified fungi, and *Purpureocillium*. The genera *Fusarium*, *Cladosporium*, *Schizothecium*, *Neomassarina*, unclassified_Nectriaceae, *Humicola*, and *Cercospora* presented relatively high relative abundances under the farmland (NT) land-use type. The genera *Staphylotrichum*, *Dactylonectria*, *Acrophialophora*, and *Talaromyces* presented relatively high relative abundances in forestland (LD). The genus *Penicillium* had higher relative abundances in nursery land (MPD) and forestland (LD) than in farmland (NT), whereas *Aspergillus* had higher relative abundances in farmland (NT) and forestland (LD) than in nursery land (MPD).

### Correlation analysis between soil physicochemical properties and the soil microbial community structure under different land use types

#### Correlation analysis of the effects of soil physicochemical properties on the soil bacterial community structure

**Fig. 5:**
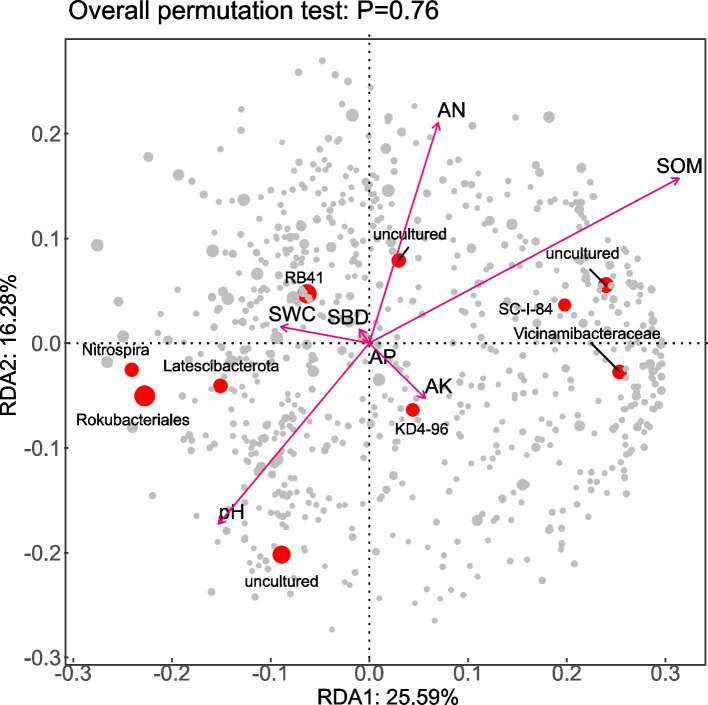
Correlation analysis between the soil physicochemical properties and bacterial community structure. Abbreviations: AK, available potassium; SWC: soil water content; pH: hydrogen potential; SBD: soil bulk density; AP, available phosphorus; AN: alkali-hydrolyzable nitrogen; SOM, total organic matter

To identify the key factors influencing soil bacterial community changes across different land use types, redundancy analysis (RDA) was performed on the soil bacterial community structure and environmental factors, including pH, available potassium (AK), available nitrogen (AN), and soil organic matter (SOM). Axis 1 explained 25.59% of the variance, and Axis 2 explained 16.28%, with a combined interpretation rate of 41.87% (Fig. 5). Axis 1 and Axis 2 reflect the effects of soil environmental factors on the differences in soil bacterial community composition at the genus level. The RDA results (Table 4) identified soil organic matter (SOM) as the key determinant of bacterial community composition at the genus level (*r*^2^ = 0.8199, *p* = 0.0040). While pH (*r*^2^ = 0.5386, *p* = 0.1054) and available nitrogen (AN; *r*^2^ = 0.5182, *p* = 0.1149) had directional effects on community structure, these relationships were not statistically significant.

**Table 4:** Effects of soil nutrients on the differences in the bacterial community at the level determined via RDA

Parameter	r^2^	*p* value
pH	0.5386	0.1054
AP	0.0005	0.9985
AK	0.1807	0.5707
AN	0.5182	0.1149
SWC	0.2128	0.5057
SBD	0.0389	0.8916
SOM	0.8199	0.0040

*AK* Available potassium, *SWC* Soil water content, *pH* hydrogen potential, *SBD* Soil bulk density, *AP* Available phosphorus, *AN* Alkali-hydrolyzable nitrogen, *SOM* total organic matter

**Fig. 6:**
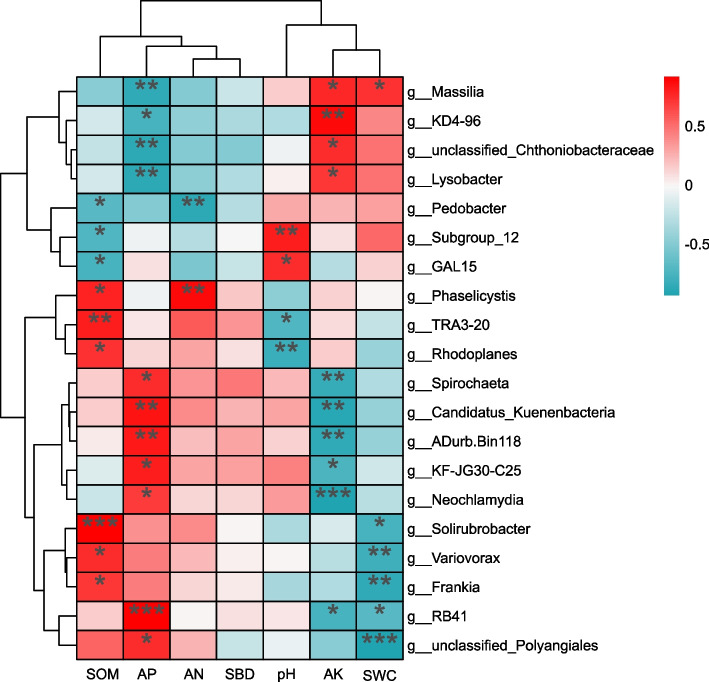
Cluster analysis of soil nutrients and bacteria in different land use types. Abbreviations: SOM, total organic matter; AP, available phosphorus; AN, alkali-hydrolyzable nitrogen; SBD, soil bulk density; pH, hydrogen potential; AK, available potassium; SWC, soil water content. A single asterisk indicates a significance value of *P < 0.05; a double asterisk indicates **P < 0.01; and a triple asterisk indicates ***P < 0.001

Through correlation analysis between the top 20 genera in terms of soil bacterial relative abundance and soil physicochemical properties under different land use types (Fig. 6), the intrinsic relationships among the composition of the soil bacterial community structure, developmental status, and soil environmental factors were further investigated. Among them, *Subgroup_12*, *RB41*, *Frankia*, and *Solirubrobacter* are assigned to Acidobacteriota; *Candidatus_Kuenenbacteria*, *Rhodoplanes*, *Variovorax*, *Massilia*, *TRA3-20*, *KF-JG30-C25*, and *Lysobacter* belong to Proteobacteria; and *Neochlamydia*, unclassified_Chthoniobacteraceae, and *ADurb. Bin118* are classified under Verrucomicrobiota; *Phaselicystis* and unclassified_Polyangiales are within Myxococcota; *Pedobacter* belongs to Bacteroidota; *KD4-96* is part of Chloroflexi; *GAL15* is assigned to p__GAL15; and *Spirochaeta* belongs to Spirochaetota. Among the soil bacteria, the genera *Massilia*, *KD4-96*, unclassified_Chthoniobacteraceae, and *Lysobacter* were significantly negatively correlated with available phosphorus (AP) but significantly positively correlated with available potassium (AK).

Conversely, the genera *Spirochaeta*, *Candidatus_Kuenenbacteria*, *ADurb. Bin118*, *KF-JG30-C25*, and *Neochlamydia* presented the opposite pattern, exhibiting a significantly positive correlation with available phosphorus (AP) and a significantly negative correlation with available potassium (AK). Additionally, *Solirubrobacter* was highly significantly positively correlated with soil organic matter (SOM), whereas *RB41* was highly significantly positively correlated with AP. Notably, *Neochlamydia* had a highly significant negative correlation with AK, and unclassified_Polyangiales exhibited a highly significant negative correlation with soil water content (SWC).

#### Correlation analysis of the effects of soil physicochemical properties on the soil fungal community structure

**Fig. 7:**
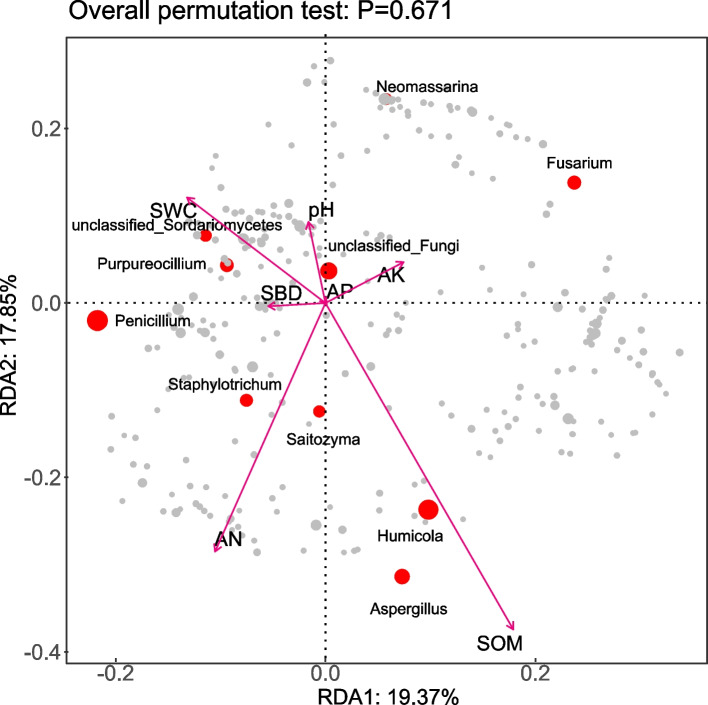
Correlation analysis between the soil physicochemical properties and fungal community structure. Abbreviations: AK, available potassium; SWC: soil water content; pH: hydrogen potential; SBD: soil bulk density; AP, available phosphorus; AN: alkali-hydrolyzable nitrogen; SOM, total organic matter

To analyze the key environmental factors influencing soil fungal community changes at the genus level across different land use types, redundancy analysis (RDA) was conducted using soil fungal communities and environmental factors, including pH, available nitrogen (AN), available potassium (AK), and available phosphorus (AP). Axis 1 accounted for 19.37% of the variance, and Axis 2 explained 17.85%, with a combined interpretation rate of 37.22%, fully reflecting the impact of soil environmental factors on soil fungal community structure changes (Fig. 7). The RDA results (Table 5) indicated that soil organic matter (SOM,* r*^2^ = 0.7430, *p* = 0.0140) was the key factor leading to changes in soil fungal communities.

**Table 5:** The impact of soil nutrients on the differences in the fungal communities at the genus level according to RDA (redundancy analysis)

Parameter	r^2^	*p* value
pH	0.1688	0.5612
AP	0.0041	0.9880
AK	0.1576	0.5977
AN	0.5441	0.1014
SWC	0.3204	0.3303
SBD	0.0983	0.7491
SOM	0.7430	0.0140

*AK* Available potassium, *SWC* Soil water content, *pH* hydrogen potential, *SBD* Soil bulk density, *AP* Available phosphorus, *AN* Alkali-hydrolyzable nitrogen, *SOM* total organic matter

**Fig. 8:**
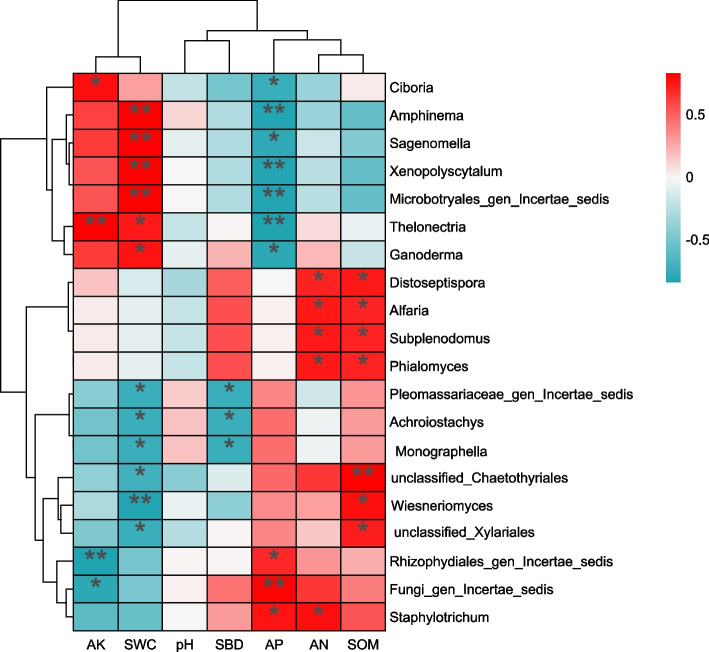
Cluster analysis of soil nutrients and fungi in different land use types. Abbreviations: AK, available potassium; SWC, soil water content; pH, hydrogen potential; SBD, soil bulk density; AP, available phosphorus; AN, alkali-hydrolyzable nitrogen; SOM, total organic matter. A single asterisk indicates a significance value of *P < 0.05; a double asterisk indicates **P < 0.01; and a triple asterisk indicates ***P < 0.001

Correlation analysis was used to investigate the intrinsic links between the top 20 genera in terms of the relative abundance of soil fungi and the soil physicochemical properties of the different land use types. Among them, *Amphinema*, *Ganoderma*, and *Microbotryales_gen_Incertae_sedis* are assigned to Basidiomycota; *Rhizophydiales_gen_Incertae_sedis* belongs to Chytridiomycota; *Fungi_gen_Incertae_sedis* is classified under Fungi_phy_Incertae_sedis; and the remaining 15 fungal genera all belong to Ascomycota. The soil water content (SWC) and available potassium (AK) were significantly correlated (*P <* 0.05/0.01) with the vast majority of fungal genera (Fig. 8). All the genera within *Basidiomycota* were significantly positively correlated with SWC. In *Ascomycota*, the genera *Subplenodomus*, *Phialomyces*, *Distoseptispora*, and *Alfaria* were significantly positively correlated with available nitrogen (AN) and soil organic matter (SOM). Additionally, pH and other environmental factors did not significantly affect the structure of the soil fungal community.

### Analysis of differences in soil microbial function among different land use types

#### Analysis of functional differences in soil bacteria

Functional prediction analysis of bacterial communities across different land use types was performed via PICRUSt2, followed by analytical annotation on the basis of the MeatCyc database. As shown in Table 6, seven primary metabolic pathways were identified across different land use types, ranked by their relative abundances as follows: biosynthesis (accounting for 67.15% ~ 68.16% of the total abundance), generation of precursor metabolites and energy (14.72% ~ 14.92%), degradation/utilization/assimilation (12.71% ~ 13.64%), metabolic clusters (2.62% ~ 2.68%), glycan pathways (0.90% ~ 0.96%), macromolecule modifications (0.74% ~ 0.77%), and detoxification (0.02% ~ 0.06%). In the biosynthesis functional pathway, forestland (LD) was significantly greater than the other land use types were (*P <* 0.05). The degradation/utilization/assimilation and detoxification metabolic pathways exhibited significant dominance in the farmland (NT) land use type (*P <* 0.05), significantly surpassing the other two land use types. The macromolecule modification metabolic pathway was significantly dominant in nursery land (MPD) (*P <* 0.05). In contrast, the generation of precursor metabolites and energy, glycan pathways, and metabolic clusters did not significantly differ among the three land use types (*P* > 0.05).

**Table 6:** Soil bacterial metabolic pathways in different land use types

Metabolic pathway	MPD	NT	LD
Biosynthesis	0.6780 ± 0.0018ab	0.6715 ± 0.0060b	0.6816 ± 0.0036a
Degradation/Utilization/Assimilation	0.1300 ± 0.0018ab	0.1364 ± 0.0050a	0.1271 ± 0.0041b
Detoxification	0.0002 ± 0.0001b	0.0006 ± 0.0003a	0.0002 ± 0.0001b
Generation of Precursor Metabolite and Energy	0.1487 ± 0.0014a	0.1492 ± 0.0023a	0.1472 ± 0.0006a
Glycan Pathways	0.0096 ± 0.0004a	0.0090 ± 0.0006a	0.0096 ± 0.0005a
Macromolecule Modification	0.0077 ± 0.0002a	0.0071 ± 0.0003b	0.0074 ± 0.0001ab
Metabolic Clusters	0.0267 ± 0.0002a	0.0262 ± 0.0006a	0.0268 ± 0.0005a

Exegesis: The data are presented as the means ± standard deviations (*n* = 3), and different lowercase letters indicate significant differences in indicator values between different land use types (*P <* 0.05). The maximum mean is marked as a. MPD stands for nursery land, NT stands for farmland, and LD stands for forestland

#### Analysis of functional differences in soil fungi

Functional prediction analysis of soil fungal community metabolism was performed via PICRUSt 2 software, and the genes were annotated on the basis of the MetaCyc database (Table 7). The results revealed that under different vegetation cover types, the primary metabolic pathways of soil fungi were primarily concentrated in five categories: biosynthesis, degradation/utilization/assimililation, generation of precursor metabolites and energy, glycan pathways, and metabolic clusters. Among these pathways, the biosynthesis metabolic pathway was the most dominant, accounting for 46.90% ~ 49.28% of the total metabolic pathways.

**Table 7:** Soil fungal metabolic pathways in different land use types

Metabolic pathway	MPD	NT	LD
Biosynthesis	0.4928 ± 0.0381a	0.4690 ± 0.0176a	0.4813 ± 0.0078a
Degradation/Utilization/Assimilation	0.1555 ± 0.0258a	0.0141 ± 0.0053a	0.1476 ± 0.0078a
Generation of Precursor Metabolite and Energy	0.2800 ± 0.0647a	0.3220 ± 0.0209a	0.3017 ± 0.0146a
Glycan Pathways	0.0141 ± 0.0012a	0.0145 ± 0.0001a	0.0136 ± 0.0003a
Metabolic Clusters	0.0576 ± 0.0022a	0.0533 ± 0.0018b	0.0558 ± 0.0006ab

Exegesis: The data are presented as the means ± standard deviations (*n* = 3), and different lowercase letters indicate significant differences in indicator values between different land use types (*P <* 0.05). The maximum mean is marked as a. MPD stands for nursery land, NT stands for farmland, and LD stands for forestland

## Discussion

### Analysis of differences in soil physical and chemical properties and microbial diversity among different land use types

Soil physicochemical properties are constrained not only by parent material and environmental conditions but are also significantly influenced by land-use practices [34, 35]. Different land-use types exert marked effects on various soil properties, including pH, soil water content (SWC), soil bulk density (SBD), and others. In this study, as shown in Table 1, the available phosphorus (AP) and available potassium (AK) contents in farmland (NT) were significantly higher than those in the other two land-use types (*P <* 0.05). Forestland (LD) also exhibited a significantly higher AP content. This pattern may be attributed to the addition of organic fertilizers resulting from artificial fertilization and crop residue retention in farmland (NT) soils [36], as well as the thicker litter layer in forestland (LD), which supplies substantial nutrients to the soil. These findings are consistent with the results reported by Qu et al. (2022) [37].

Changes in soil microbial community diversity indices profoundly influence soil physicochemical properties and plant growth and development [38]. Different land-use practices directly affect soil physicochemical properties, thereby impacting the development of soil microbial community structure [39, 40]. Variations in microbial community diversity indices are of critical importance for evaluating soil health and ecosystem stability [41, 42]. This study found (Table 2) that among soil bacterial community diversity indices, the Simpson index in farmland (NT) showed a significant advantage (*P <* 0.05), consistent with the results reported by Liu et al. (2022) [34]. This may be attributed to the favorable soil physicochemical conditions in farmland (NT), which provide a suitable habitat for soil microorganisms. For soil fungal community diversity indices, both farmland (NT) and forestland (LD) exhibited significant advantages in the Shannon and Simpson indices (*P <* 0.05), but the diversity indices were higher in farmland (NT) than in forestland (LD), which differs from the findings reported by Zhu et al. (2022) [43]. This discrepancy may be because forestland (LD) experiences less external disturbance and possesses richer organic matter inputs, thereby forming a more stable soil environment conducive to the development of fungal diversity.

### Analysis of soil microbial communities in different land use types

Soil environmental factors are directly related to and exert substantial influences on soil microbial communities [44]. Redundancy analysis (RDA) of the relationships between genus-level species composition of bacteria and fungi and soil physicochemical properties under three different land-use types (Figs. 4, 6) revealed that soil organic matter (SOM) was the key factor affecting the development of soil microbial community structure (*P <* 0.05). This finding differs from the results reported by Zhang et al. [10] and Wang et al. [18], who identified available phosphorus (AP) and pH as the key factors influencing microbial community structure. The discrepancy may be due to the transitional climate and diverse land-use patterns in the Huaihe River source region, which amplify the critical ecological role of SOM as both a carbon source and a microhabitat regulator [45]. Further correlation analysis between soil environmental factors and the top 20 dominant genera of bacteria and fungi by relative abundance (Figs. 5, 7) showed that among the bacterial genera, 11 were significantly correlated with AP and 10 with available potassium (AK). For fungi, 7 of the top 20 dominant genera were significantly correlated with SOM, while AP and soil water content (SWC) showed significant correlations with 10 and 12 genera, respectively. Additionally, 9 genera exhibited significant or highly significant correlations with SOM. These results indicate that soil environmental conditions substantially influence the structure and composition of soil microbial communities, which aligns with findings from Zhou et al. [46] and Wang et al. [47].

### Analysis of soil microbial community structure under different land-use types

Analysis of relative bacterial phylum abundances revealed (Figs. 2 and 3) that the dominant bacterial phyla across the three land-use types were Acidobacteria, Proteobacteria, and Actinobacteria, collectively accounting for over 55% of the relative abundances of the top ten dominant phyla across all land-use types. These three phyla constitute dominant groups in most soil bacterial communities, consistent with prior research [3, 48]. The dominance of Acidobacteria and Proteobacteria facilitates the decomposition of soil organic matter and the cycling of elements such as carbon, nitrogen, and sulphur within the soil, thereby ensuring the availability of nutrients essential for plant growth and development [49]. This also indicates that all three land use types possess active potential for organic matter decomposition and nutrient transformation, which is conducive to maintaining soil fertility and ecological functions. Among the top 20 dominant genera in bacterial relative abundance across land use types, the Acidobacteria phylum included genera such as *RB41*, *Subgroup_2*, *uncultivated* genera, and *Vicinamibacteraceae*; the Chlorobactaceae phylum featured *uncultivated* genera and *KD4-96*, demonstrating significant dominance. These bacterial communities profoundly influence the physicochemical properties and bacterial development within soils across different land use types.

In fungi, Ascomycota was the dominant phylum, constituting over 77% of the total relative abundance. Most Ascomycota fungi are saprophytic and thrive within a pH range of 7–8 [50]. The relatively higher dominance of Ascomycota in farmland (NT) reflects an ecosystem characterized by abundant crop‑residue resources and frequent anthropogenic disturbance. This also suggests the need to monitor potential pathogen accumulation that may affect crop health, while indicating that fungal microorganisms in farmland (NT) contribute to the degradation of plant residues in soil [51]. Genus‑level analysis of the soil fungal community revealed that 18 of the top 20 dominant fungal genera belonged to Ascomycota, with the genus *unclassified_Fungi* assigned to the phylum *unclassified_Fungi* and the genus *Saitozyma* belonging to Basidiomycota. The dominance of these fungal genera varied across different land‑use types, leading to differential impacts on soil material cycling, ecosystem stability, and the development of soil environmental quality.

### Analysis of metabolic pathways in soil microbial communities across different land use types

Functional prediction analysis of microbial communities is crucial for understanding the role of soil microbial communities in soil processes [19, 52]. Differences in soil microbial community functions determine the direction of regulation and evolution within soil ecosystems [53]. Analysis of bacterial and fungal metabolic pathways in soil (Tables 6 and 7) reveals that the Biosynthesis pathway constitutes the predominant metabolic pathway within both bacterial and fungal communities. This indicates that the Biosynthesis pathway plays a pivotal role in the conversion of inorganic substances into organic matter by these microbial communities. These organic materials constitute an essential component of soil fertility, being vital for maintaining soil structure and function while providing the material basis and energy source for other organisms within the soil.

## Conclusion

This study indicates that land use patterns have a significant impact on soil physicochemical properties and microbial community structure (*P <* 0.05). Compared with forestland and nursery land, farmland performs better in some nutrient indicators and microbial diversity, which suggests that moderate agricultural cultivation can maintain a relatively high level of underground microbial diversity to a certain extent. The results of community structure composition show that different land use types have their own dominant groups, but under the constraints of similar environmental conditions, the community structure may still present a certain convergent pattern, reflecting the important role of environmental filtering in community construction. Redundancy analysis (RDA) further confirms that soil organic matter (SOM) is a key factor driving the differences in bacterial and fungal community composition (*P <* 0.05); functional prediction results show that biosynthesis-related pathways are the main metabolic functional modules of bacteria and fungi. In general, the research results emphasize the coupling relationship between "land use—soil organic matter—microbial community structure/function", and provide a microbial ecological basis for improving soil health and optimizing land use in the source area of the Huaihe River. However, the sample plots and land use types in this study are relatively concentrated. This study is based on one-time sampling with limited repetitions and mainly focuses on the 0–30 cm surface soil. Future research can be improved through multi-seasonal and multi-year monitoring and sampling of deeper soil layers; the microbial function results are mainly derived from PICRUSt2 predictions. In the future, further research can be conducted by combining metagenomics, transcriptomics, functional genes, enzyme activities and other measured methods.

## Data Availability

The data and figures used in this study are included in the main text or in the Supporting Information. The microbial data have been uploaded to the NCBI database, and the serial number is BioProject: PRJNA1233557.

## Abbreviations

MPD: Nursery land
NT: Farmland
LD: Forestland
SOM: Soil organic matter
AN: Alkaline hydrolysis nitrogen
AP: Available phosphorus
AK: Available potassium
SWC: Soil moisture content
SBD: Soil bulk density
